# Mouse Adapted SARS-CoV-2 (MA10) Viral Infection Induces Neuroinflammation in Standard Laboratory Mice

**DOI:** 10.3390/v15010114

**Published:** 2022-12-30

**Authors:** Narayanappa Amruta, Saifudeen Ismael, Sarah R. Leist, Timothy E. Gressett, Akhilesh Srivastava, Kenneth H. Dinnon, Elizabeth B. Engler-Chiurazzi, Nicholas J. Maness, Xuebin Qin, Jay K. Kolls, Ralph S. Baric, Gregory Bix

**Affiliations:** 1 Department of Neurosurgery, Clinical Neuroscience Research Center, Tulane University School of Medicine, New Orleans, LA 70112, USA; 2 Department of Epidemiology, Microbiology and Immunology, University of North Carolina at Chapel Hill, Chapel Hill, NC 27599, USA; 3 Tulane Brain Institute, Tulane University, New Orleans, LA 70112, USA; 4 Center for Translational Research in Infection and Inflammation, Tulane University School of Medicine, New Orleans, LA 70112, USA; 5 Department of Neurology, Tulane University School of Medicine, New Orleans, LA 70112, USA; 6 Department of Microbiology and Immunology, Tulane University School of Medicine, New Orleans, LA 70112, USA; 7 Tulane National Primate Research Center, Covington, LA 70433, USA; 8 Tulane University School of Public Health and Tropical Medicine, New Orleans, LA 70122, USA

**Keywords:** animal models, brain, COVID-19, mouse-adaptation, neuroinflammation, SARS-CoV-2

## Abstract

Increasing evidence suggests that severe acute respiratory syndrome coronavirus 2 (SARS-CoV-2) infection impacts neurological function both acutely and chronically, even in the absence of pronounced respiratory distress. Developing clinically relevant laboratory mouse models of the neuropathogenesis of SARS-CoV-2 infection is an important step toward elucidating the underlying mechanisms of SARS-CoV-2-induced neurological dysfunction. Although various transgenic models and viral delivery methods have been used to study the infection potential of SARS-CoV-2 in mice, the use of commonly available laboratory mice would facilitate the study of SARS-CoV-2 neuropathology. Herein we show neuroinflammatory profiles of immunologically intact mice, C57BL/6J and BALB/c, as well as immunodeficient (*Rag2*^−/−^) mice, to a mouse-adapted strain of severe acute respiratory syndrome coronavirus-2 (SARS-CoV-2 (MA10)). Our findings indicate that brain IL-6 levels are significantly higher in BALB/c male mice infected with SARS-CoV-2 MA10. Additionally, blood-brain barrier integrity, as measured by the vascular tight junction protein claudin-5, was reduced by SARS-CoV-2 MA10 infection in all three strains. Brain glial fibrillary acidic protein (GFAP) mRNA was also elevated in male C57BL/6J infected mice compared with the mock group. Lastly, immune-vascular effects of SARS-CoV-2 (MA10), as measured by H&E scores, demonstrate an increase in perivascular lymphocyte cuffing (PLC) at 30 days post-infection among infected female BALB/c mice with a significant increase in PLC over time only in SARS-CoV-2 MA10) infected mice. Our study is the first to demonstrate that SARS-CoV-2 (MA10) infection induces neuroinflammation in laboratory mice and could be used as a novel model to study SARS-CoV-2-mediated cerebrovascular pathology.

## 1. Introduction

Since its emergence in late 2019, SARS-CoV-2 has been a significant source of morbidity and mortality worldwide. It is now known that infection can cause a wide range of mild to severe symptoms, especially in patients with significant comorbidities such as chronic lung disease. Importantly, those with cerebrovascular diseases, including brain ischemia and vascular dementia, are more susceptible to SARS-CoV-2 systemic infection [1,2,3,4], suggesting important consequences for the central nervous system (CNS). Indeed, recent autopsy cases of Coronavirus Disease 2019 (COVID-19) brains propose that SARS-CoV-2 can access the CNS through axonal transport or viral migration through neurons in the brain [5,6,7]. In this way, SARS-CoV-2 may enter the CNS through both the olfactory bulb and normal blood circulation [8]. Intriguingly, patients hospitalized with COVID-19 and who have concurrent neurological problems have a higher risk of mortality than other patients, although the exact pathophysiology remains elusive and additional studies are required to fully explore CNS-mediated COVID-19 complications [1,2,9].

Given the prominence of acute and chronic neurologic sequelae, a suitable animal model that mimics human SARS-CoV-2 infection and its associated brain disease are of paramount importance. Several mouse models have been developed to support susceptibility to SARS-CoV-2 infection via the expression of human ACE-2 (hACE-2), including K18-hACE2, knock-in strains, as well as viral vector-mediated delivery of hACE2. These models have provided many important insights regarding the consequences of SARS-CoV-2 infection. However, these models are not ideal for evaluating neurological consequence that mimics human SARS-CoV-2 infection and its associated brain disease [1,10,11,12]. The hACE-2 K18 transgenic mouse model, for example, relies on expressing hACE-2 in K18+ epithelial cells, which ultimately are not adequate for neuropathological study as they do not mimic the natural expression pattern of the ACE-2 receptor [10]. Additional models which rely on lentivirus-generated SARS-CoV-2 pseudovirus or adenovirus delivery of hACE-2 have shown promising results but also suffer from similar limitations [12,13,14,15]. 

Recently, a Mouse Adapted strain of severe acute respiratory syndrome coronavirus-2 (SARS-CoV-2 (MA10)) infection model in mice was reported to infect standard laboratory female mice (C57BL/6J and BALB/c) and has emerged as a promising candidate to study multiple aspects of SARS-CoV-2 disease pathogenesis [16,17]. As previously published, a mouse-adapted SARS-CoV-2 (MA10) induces acute lung injury and mortality with no detectable virus found in the brains of the infected BALB/c mice at the time of peak lung titer 2 days post-infection (2 dpi), indicating restricted neurotropism of SARS-CoV-2 in laboratory mice. However, the neurological consequences of SARS-CoV-2 (MA10) exposure are not yet known. In the present study, our main objective was to determine the extent to which SARS-CoV-2 (MA10) infection in C57BL/6J, BALB/c and immunodeficient (*Rag2*^−/−^, lacking mature lymphocytes due to inability to initiate V(D)J rearrangement [18] mice impacts the CNS. We hypothesized that exposure to SARS-CoV-2 would impact brain function. Using intranasal inoculation with SARS-CoV-2 (MA10) as a model of CNS effects of COVID-19, we, therefore, evaluated the impact of SARS-CoV-2 infection on acute and chronic neurovascular integrity and neuroinflammation. Our results demonstrate that acute SARS-CoV-2 infection results in significant and prolonged acute and chronic neurological sequelae.

## 2. Materials and Methods

### 2.1. Mice and Ethics Statement

Male 10-12-week-old mice were obtained from The Jackson Laboratory. *Rag2^−/−^* mice on a C57BL/6 background have a disruption of the recombination activating gene 2 (*Rag2*) and fail to produce mature T or B lymphocytes [19]. Mice were housed in the animal facility at Tulane University School of Medicine. The Institutional Animal Care and Use Committee of Tulane University reviewed and approved all procedures for sample handling, inactivation, and removal from a BSL3 containment (permit number 4267). One-year-old female BALB/cAnNHsd mice (strain 047) (herein referred to as “BALB/c” female mice throughout the manuscript) were obtained from Envigo and were housed at the University of North Carolina at Chapel Hill.

### 2.2. SARS-CoV-2 Infection 

Male mice were inoculated with either mock or SARS-CoV-2 (MA10) strain of severe acute respiratory syndrome coronavirus 2 (SARS-CoV-2, BEI Resources, NR-55329) in a total volume of 50 µL via intranasal administration by ABSL3-trained staff with a dose of 1 × 10^5^ TCID_50_/mouse to induce viral infection in these animals. Infected mice were observed daily for changes in body weight and clinical signs of illness. After 3 days (3 dpi), the mice were euthanized by CO_2_ asphyxiation followed by cervical dislocation. The lungs and brains were collected. The right hemisphere was harvested for histology and immunofluorescence, and the left hemisphere for gene expression analysis. One-year-old female BALB/c mice were intranasally infected with 10^3^ PFU SARS-CoV-2 (MA10) under BSL3 conditions at the University of North Carolina at Chapel Hill. Whole brains were harvested for histology and immunofluorescence.

### 2.3. Gene Expression

Tissues were homogenized in Trizol Lysis Reagent, and RNA was extracted from the left hemisphere of the brain according to RNA extraction kit manufacturer instructions (RNeasy Plus Mini Kit; Qiagen). RNA was converted to cDNA using iScript reverse transcriptase master mix (Bio-Rad, Hercules, CA, USA). Gene expression was carried out with QuantStudio 3 Real-Time PCR Systems (Life Technologies, Carlsbad, CA, USA) using TaqMan PCR Master Mix and premixed primers/probe sets (Thermo Fisher Scientific, Waltham, MA, USA) specific for *Il-1β* (Mm00434228_m1), *IL-6* (Mm00446190_m1), *Ocel1* (Mm01349279_m1), *Cldn5* (Mm00727012_s1), *GFAP* (Mm01253033_m1), *Tnf-α* (Mm00443258_m1), *Ccl2* (Mm00441242_m1), *Cxcl10* (Mm00445235_m1), and *Gapdh* (Mm99999915_g1, Control gene) (Life Technologies) for gene expression [20]. Data was analyzed comparing control to SARS-CoV-2 infected mice and are presented as a fold change of control. Subgenomic mRNA encoding the N gene (sgm-N) was quantified using a published assay [20]. The viral copy numbers from the lung samples are represented as copies/100 ng of RNA [21].

### 2.4. Histology

The tissue of one-year-old female BALB/c mice intranasally infected with 10^3^ PFU of mouse-adapted SARS-CoV-2 (MA10) or mock-infected [16] were stored in 10% phosphate-buffered formalin fixative and processed for paraffin embedding. After paraffin embedding, paraffin sections (5 μm in thickness) were used for hematoxylin and eosin (H&E) stains to identify morphological changes in brains. Slides were scanned with a digital slide scanner (Zeiss Axio Scan; Zeiss, White Plains, NY, USA). Representative photomicrographs at 20× magnification were acquired from whole scanned coronal sections using the Aperio Image Scope software (version 12.3.2.8013, Leica, Buffalo Grove, IL, USA). The number of perivascular cuffing sites was counted from all the mice in each group (uninfected control n = 5; SARS-CoV-2 (MA10)-infected n = 5) and was quantified by a pathological score. Whole specimens were examined by using Aperio Image Scope software to establish a histopathological score in each case.

### 2.5. SARS-CoV-2 Immunofluorescence

Immunofluorescent analysis was conducted on the brains of one-year-old female BALB/c mice intranasally infected with 10^3^ PFU of mouse-adapted SARS-CoV-2 MA10 or mock infection (detailed experimental protocol as previously described [16]). Paraffin sections (5 μm in thickness) were used, and slides were deparaffinized in xylene and rehydrated through an ethanol series, followed by heat-induced antigen retrieval with high pH antigen unmasking EDTA solution. The slides were washed with PBS with 0.3% Triton X-100 and blocked with 5% normal goat serum for 1 h. Primary antibodies include anti-Iba1, Rabbit (1:1000, Cat# 019-19741, FUJIFILM Wako Pure Chem Corp), and glial fibrillary acidic protein (GFAP) Polyclonal Antibody (1:1000, Cat# PA5-16291, Invitrogen) incubation was achieved at room temperature for 1 h. Slides were then washed, and the primary antibody was detected following 60 min incubation in an appropriate secondary antibody tagged with Alexa Fluor fluorochromes (1:1000) in normal goat serum. After washing in PBS, mounting media with DAPI was used to label the nuclei.

### 2.6. Statistics

Statistical tests were performed using GraphPad Prism, 9.3.1 version (GraphPad Software, San Diego, CA). Data are presented as mean ± SEM. Significant differences were designated using omnibus one-way ANOVA and, when significant, followed up with two-group planned comparisons selected *a priori* to probe specific hypothesis-driven questions (saline vs SARS-CoV-2MA10). Statistical significance was taken at the *p* < 0.05 level. 

## 3. Results

### 3.1. Effect of Severe Acute Respiratory Syndrome Coronavirus-2 (MA10) Infection on Subgenomic N (Sgm-N) Viral Load in Laboratory Mice

**Figure 1:**
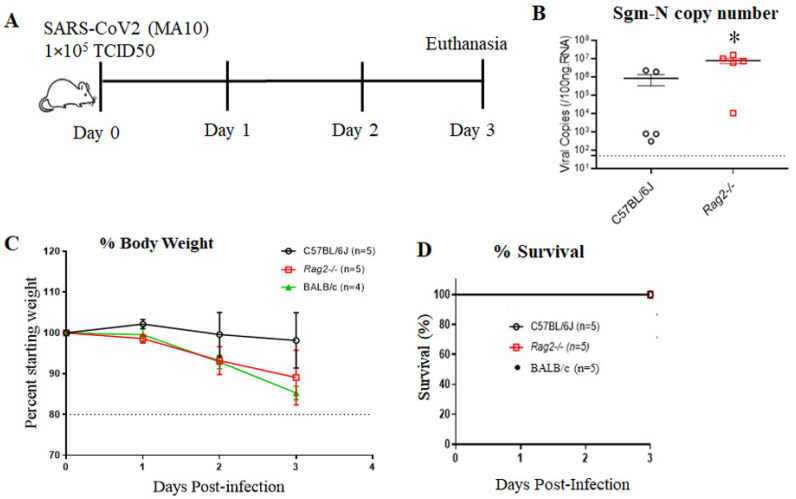
C57BL/6J mice show less disease severity compared to BALB/c and *Rag2*^−/−^ mice following mouse-adapted SARS-CoV2 (MA10) infection. (**A**) Schematic overview of the experimental timeline for 10-week-old male C57BL/6, BALB/c, and *Rag2*^−/−^ mice. Animals were inoculated via the intranasal route with mock (PBS) or MA10 strain of SARS-CoV-2 (1 × 10^5^ TCID_50_). Upon 3 days post-infection (3 dpi), mice were euthanized, and RNA isolated from the lung samples by the Trizol method was processed for gene expression. (**B**) Subgenomic-N viral copies and (**C**) Percent starting weight loss in SARS-CoV-2 (MA10)-infected C57BL/6 and SARS-CoV-2 (MA10) infected *Rag2*^−/−^ mice and BALB/c (**D**) Survival rate. Data were analyzed using 2-factor ANOVA followed by Sidak’s multiple comparisons if more than two groups. The Mann-Whitney U test is used to compare the difference in N viral copies between C57Bl/6J and *Rag2^−/−^*. Error bars represent mean +/− SEM, * *p* < 0.05, n = 4–5/group.

10 12-week-old male C57BL/6J, BALB/c and *Rag2*^−/−^ mice were inoculated via the intranasal route with mock or SARS-CoV-2 (MA10) strain of SARS-CoV-2 (1 × 10^5^ TCID_50_) (Figure 1A). At 3 days post-infection (3 dpi), the mice were euthanized, and RNA was isolated from half of the left lung by the Trizol method for gene expression. Lung tissue expression of viral load after 3 dpi with SARS-CoV-2 (MA10) was significantly higher in the lungs of *Rag2*^−/−^ (8.0 ± 6.0 × 10^6^ copies/100 ng RNA) compared to C57BL/6J (8.6 ± 11.7 × 10^5^ copies/100 ng RNA) infected mice (Figure 1B). Interestingly, we did not detect the virus in the brains of the MA10-infected mice at 3 dpi, the data is added as Supplementary Figure S1. In comparison to C57BL/6J, 10-week-old BALB/c and *Rag2*^−/−^ mice exhibited more disease and associated weight loss (up to 10–20%), but no mortality after infection in all three groups was observed (Figure 1C,D).

### 3.2. Cytokine and Chemokine Responses in the Brains of Mouse Adapted Strain of SARS-CoV-2 (MA10) Infected Laboratory Mice

**Figure 2:**
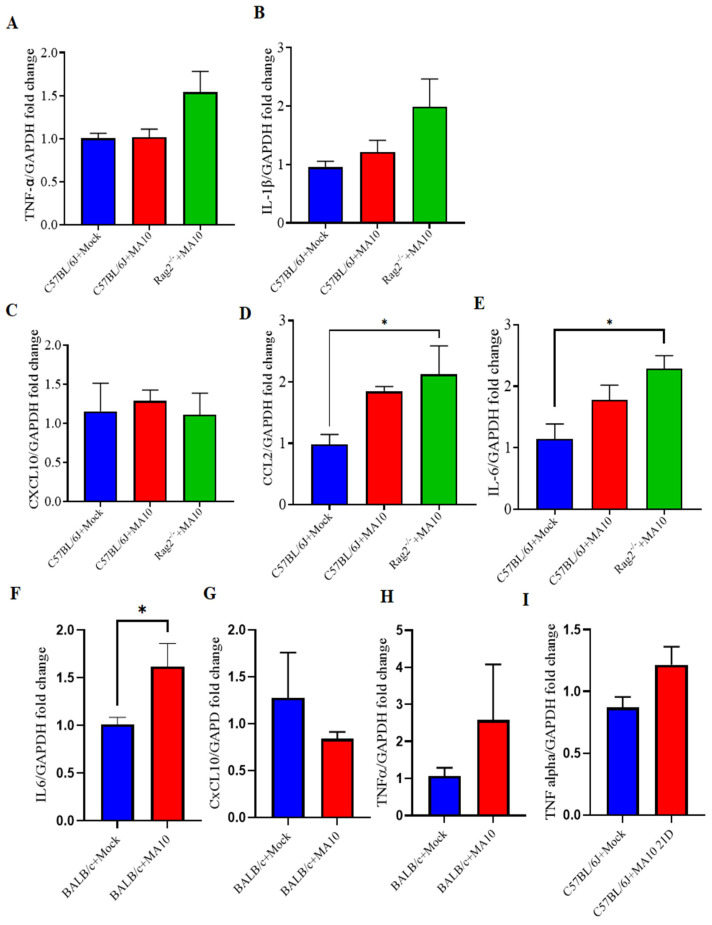
CNS cytokine and chemokine responses in the brains of mouse-adapted SARS-CoV-2 (MA10) infected laboratory mice. 10-week-old male C57BL/6, BALB/c and *Rag2*^−/−^ mice were inoculated via the intranasal route with mock or MA10 strain of SARS-CoV-2 (1 × 10^5^ TCID_50_). Upon 3 days post-infection (3 dpi), mice were euthanized, and RNA was isolated from the left hemisphere for gene expression analysis. (**A**) *TNF-α* and (**B**) *IL-1β* mRNA expression in brains of SARS-CoV-2 (MA10) infected C57BL/6J mice did not significantly differ from those of mock-infected mice. (**C**) *Cxcl10*; (**D**) *Ccl2* and (**E**) *IL-6* expression in brains of SARS-CoV-2 (MA10) infected *Rag2*^−/−^ mice and C57BL/6J mock-treated mice. (**F**) *IL-6* (**G**) *CxCL10* (**H**) *TNF-α* expression in BALB/c mice. (**I**) 21 days post-infection (21 dpi), male mice were euthanized and analyzed for *TNF-α* mRNA expression in C57BL/6 mice, SARS-CoV-2 MA10 infected C57BL/6 mice show a trend towards increased *TNF-α* expression compared to mock-treated mice. IL-6 was significantly increased, and TNF-α showed an upward trend in SARS-CoV-2 MA10-infected BALB/c mice compared with mock-treated mice. Data are presented as mean ± SEM. *p* values represent mock vs. SARS-CoV-2 (MA10) challenged groups. Significant differences are designated using a two-tailed unpaired student t-test for two groups and one-way ANOVA (for more than two groups). Some of the experiment n = 4 indicates 2 mice were excluded because the dissection of half left hemisphere did not align with other mice. C57BL/6J + Mock n = 5; C57BL/6J + SARS-CoV-2 MA10 n = 4; BALB/c + Mock n = 4–5; BALB/c + SARS-CoV-2 MA10 n = 4–5; *Rag2*^−/−^ + SARS-CoV-2 MA10 n = 4; * *p* < 0.05.

C57BL/6J and BALB/c are the most commonly used background strains for the majority of transgenic mice [22]. The mRNA expression of *TNF-α* (Figure 2A), *IL-1β* (Figure 2B), and *Cxcl10* (Figure 2C) were not different in the brains of SARS-CoV-2 MA10-infected male C57BL/6J mice. The mRNA expression level of IL-1β and TNFα has shown an increasing trend in *Rag2^−/−^* mice following SARS-CoV-2 MA10 infection. However, *IL-6* (Figure 2E) was significantly increased in SARS-CoV-2 (MA10)-infected *Rag2*^−/−^. Whereas *IL-6* (Figure 2F) was significantly increased, *TNF-α* (Figure 2H) showed an upward trend and no difference in *Cxcl10* (Figure 2G) in SARS-CoV-2 MA10-infected BALB/c mice compared with the mock-infected group. Relative to C57BL/6J mock-treated mice, levels of *Ccl2* expression were significantly higher in brains of SARS-CoV-2 (MA10)-infected *Rag2*^−/−^, but not in SARS-CoV-2 (MA10)-infected C57BL6, male mice as (Figure 2D); *Ccl2* expression levels between SARS-CoV-2 (MA10)-infected C57BL/6 and *Rag2*^−/−^ mice did not differ from each other.

### 3.3. Claudin-5 mRNA Expression Is Decreased, and GFAP Expression Increased in the Brains of Mouse Adapted Strain of SARS-CoV2 (MA10) Infected Mice

**Figure 3:**
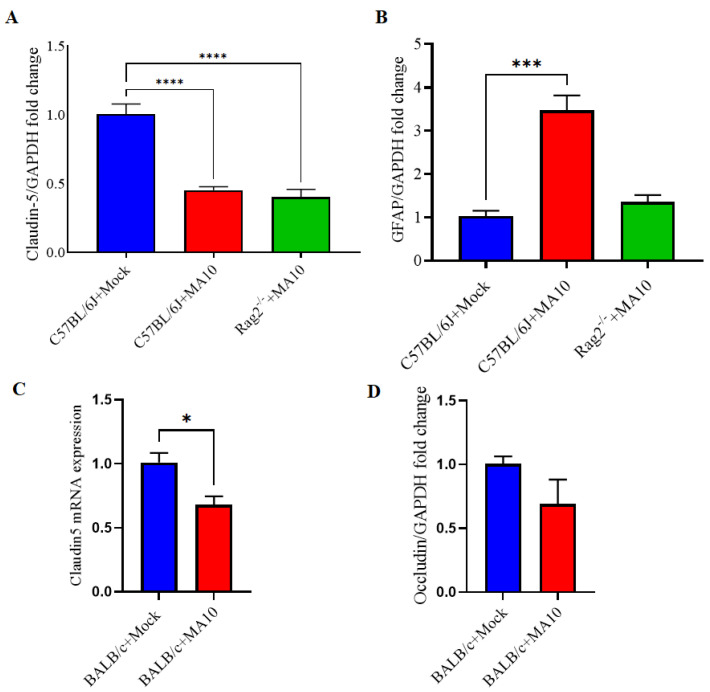
mRNA expression of Claudin-5 is decreased, and GFAP increased in the brains of mouse-adapted SARS-CoV-2 (MA10) infected mice. 10-week-old male C57BL/6J, BALB/c and *Rag2*^−/−^ mice were inoculated via the intranasal route with mock or MA10 strain of SARS-CoV-2 (1 × 10^5^ TCID_50_; Red bar). Upon 3 days post-infection (3 dpi), mice were euthanized, and RNA was isolated from the half-left hemisphere of the brain by the Trizol method for gene expression analysis. (**A**) Claudin-5 and (**B**) GFAP mRNA expression in brains of mock or SARS-CoV-2 (MA10) infected C57BL/6 mice, whereas (**C**) Claudin-5 (**D**) Occludin expression in BALB/c mice were evaluated. Claudin-5 expression was significantly lower in brains of all SARS-CoV-2 (MA10) infected C57BL/6J, BALB/c and *Rag2*^−/−^ mice compared with C57BL/6J and BALB/c mock-treated mice. GFAP expression was significantly higher in the brains of SARS-CoV-2 (MA10) infected C57BL/6J mice. Occludin expression (**D**) showed a downtrend in SARS-CoV-2 MA10-infected BALB/c mice compared with mock-treated mice. Data are presented as mean ± SEM. *p* values represent mock vs. SARS-CoV-2 (MA10) challenged groups. Significant differences are designated using a two-tailed unpaired student t-test for two groups and one-way ANOVA (for more than two groups). C57BL/6J + Mock n = 5; C57BL/6J + SARS-CoV-2 MA10 n = 5; *Rag2*^−/−^ + SARS-CoV-2 MA10 n = 5; * *p* < 0.05, *** *p* < 0.001, **** *p* < 0.0001.

Claudin-5 expression as a measure of blood-brain barrier integrity was significantly lower in brains SARS-CoV-2 (MA10) infected C57BL/6J and *Rag2*^−/−^ (Figure 3A), BALB/c (Figure 3C), male mice compared with mock-treated groups. There is a downward trend in occludin mRNA in BALB/c mice following infection (Figure 3D). Additionally, GFAP expression as a measure of resident immune response was significantly higher in the brains of SARS-CoV-2 (MA10) infected C57BL/6J mice (Figure 3B).

### 3.4. SARS-CoV-2 (MA10) Infection Significantly Increases Iba-1 Positive Microglial Cells in Cortical Region of Brain of 1-Year Old Female BALB/c Mice

**Figure 4:**
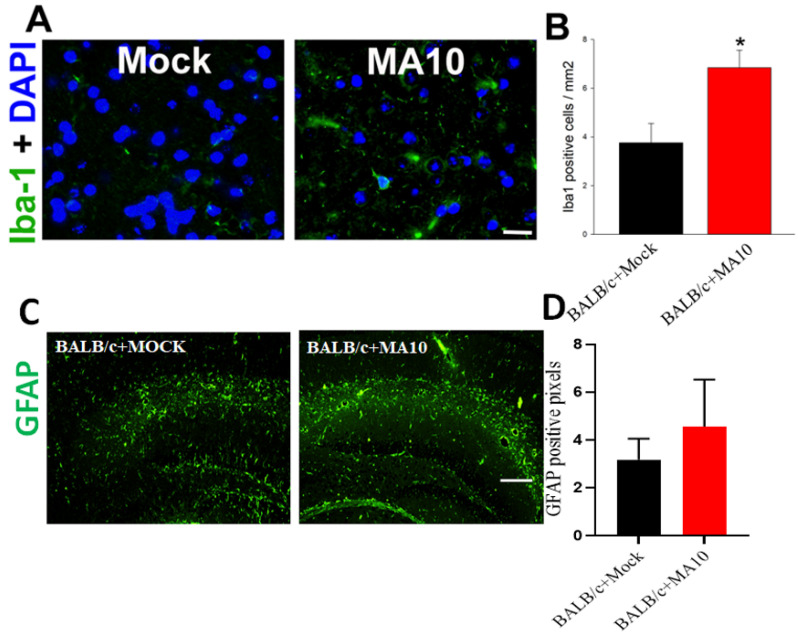
SARS-CoV-2 (MA10) infection significantly increases Iba-1 positive microglial cells in the cortex region of the brain in 1-year-old female BALB/c mice. 1-year-old female BALB/c mice were inoculated via the intranasal route with saline (black bar) or SARS-CoV-2 (MA10) strain of SARS-CoV-2 (1 × 10^3^ PFU/mL) (Red bar) (Detailed experimental methods are available in Dinnon et al., 2020; Leist et al., 2021). Upon 2 days post-infection (2 dpi), mice were euthanized, and the whole brain was harvested and fixed in 10% phosphate-buffered formalin, paraffin-embedded and sectioned at 4μm thickness. Sequential sections were stained with Iba-1 by immunofluorescence. (**A**) Representative images of Iba-1 positive microglial cells (Iba1 staining, green) with DAPI as nuclear counterstaining show high induction in the cortex region of brains of SARS-CoV-2 (MA10) infected female BALB/c mice. (**B**) Quantification of Iba-1 positive microglial cells was significantly higher in SARS-CoV-2 (MA10) infected brains compared with mock infection. SARS-CoV-2 (MA10) infection increases (**C**,**D**) GFAP-positive cells in the hippocampus of 12-week old male BALB/c mice after 3 days of infection. Data are presented as mean ± SEM. *p* values represent saline vs SARS-CoV-2 (MA10) challenged groups. Significant differences are designated using a two-tailed unpaired student t-test. Saline n = 5; SARS-CoV-2 MA10 n = 6, * *p* < 0.01. Scale bar: 40× magnification, scale bar  =  25 μm.

1-year-old female BALB/c mice were inoculated intranasally with either saline (black bar) or SARS-CoV-2 (MA10) (1 × 10^3^ PFU/mL) (red bar) as described previously [16,23]. A previous study reported that there is no detectable virus found in the brains of the infected BALB/c mice at the time of peak lung titer 2 days post-infection (2 dpi) [16]. At 2 dpi, the mice were euthanized, and the whole brain was harvested and fixed in 10% phosphate-buffered formalin, followed by embedding in paraffin and sectioning at 4 um thickness. Sequential sections were stained with Iba-1 by immunofluorescence. Representative images of Iba-1 positive microglial cells (Iba1 staining, green) with DAPI as nuclear counterstaining showed that Iba1 levels are highly induced in the cortical region of brains of SARS-CoV-2 (MA10) infected female BALB/c mice (Figure 4A). Quantification of Iba-1 positive microglial cells was significantly higher in SARS-CoV-2 (MA10) infected brains compared with mock infection (Figure 4B). SARS-CoV-2 (MA10) infection showed a trend towards increased *GFAP* expression in the hippocampus of 12-week old male BALB/c mice after 3 days of infection compared to mock-treated mice (Figure 4C,D).

### 3.5. SARS-CoV-2 (MA10) Infection Increases Perivascular Lymphocyte Cuffing in Brains of 1-Year-Old Female BALB/c

**Figure 5:**
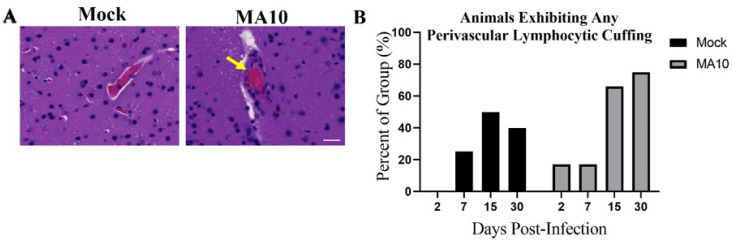
SARS-CoV-2 (MA10) infection increases perivascular lymphocyte cuffing in the brains of BALB/c mice. 1-year-old female mice were infected as in Figure 3. (**A**) Representative H&E-stained brain images as labeled demonstrate significant perivascular lymphocyte cuffing (yellow arrowheads) at 30 days (30 dpi) post-infection SARS-CoV-2 (MA10) infected female mice. Scale bar = 20 μm. (**B**) Quantification of the percent of each group of animals (Saline n = 4; MA10 n = 6) with any detectable perivascular lymphocyte cuffing at various days post-infection as labeled. A total of saline n = 16 and MA10 n = 24 were included in the study. Saline n = 4; MA10 n = 6 was euthanized at each time point (each group of animals), and all mice were undergone for histological analysis. Graphs represent only the percent of each group of animals that showed positive for histopathological symptoms of perivascular lymphocyte cuffing. Chi-Square trend analysis shows a significant change in the portion of mice with detectable perivascular lymphocytic cuffing over time only in SARS-CoV-2 (MA10)-infected mice.

Representative H&E-stained brain images demonstrate prominent perivascular lymphocyte cuffing (an indicator of vascular inflammation, yellow arrowheads) at 30 days post-infection (30 dpi) in SARS-CoV-2 (MA10) infected female mice (Figure 5A). Quantification of the percent of each group of animals (Saline n = 4; SARS-CoV-2 (MA10) n = 6) with any detectable perivascular lymphocyte cuffing at 30 days post-infection were performed as labeled. Chi-Square trend analysis of the proportion of mice displaying detectable perivascular lymphocyte cuffing across time (dpi 2, 7,15, and 30) in each group separately (mock- vs SARS-CoV-2 (MA10)-infected) was significant only in SARS-CoV-2 (MA10)-infected mice [χ^2^ trend: 5.41, *p* = 0.020] (Figure 5B); no such change was detected in mock-infected mice. Visual inspection of the graph indicates that the proportion of SARS-CoV-2 (MA10)-infected mice displaying perivascular lymphocyte cuffing increased as time progressed.

## 4. Discussion

Suitable animal models for the study of SARS-CoV-2 pathophysiology are important tools in the development of novel therapeutics and in understanding fundamental biological mechanisms of how COVID-19 may affect neurological function over time. As the full extent of the neuropathological changes induced by SARS-CoV-2 is currently unknown, with limited availability of relevant animal models, we sought to characterize the pathological changes induced in the brain after acute and chronic infection with a novel mouse-adapted SARS-CoV-2 (MA10) in the standard laboratory or immunocompromised mice. As this model has been shown to recapitulate disease course and severity in mice comparable to that in humans [16,24], we analyzed key markers in the brain relevant to immune and vascular function after successful viral inoculation.

The study demonstrated that SARS-CoV-2 (MA10) infection did not cause any mortality in 10-week-old C57BL/6, BALB/c, and *Rag2*^−/−^ mice, although there is an increase in pulmonary viral load and increased weight loss in C57BL/6, BALB/c and *Rag2*^−/−^ mice. Consistently, we have previously shown elevated pulmonary viral load in BALB/c mice following MA10 infection [16]. However, Leist et al. reported that SARS-CoV-2 (MA10) infection causes mortality in BALB/c and C57BL/6 mice in an age-dependent manner [16]. Quantitative real-time PCR analysis of key inflammatory mediators demonstrated that SARS-CoV-2 (MA10) infection elevated cytokine *IL-6* in BLAB/c and chemokine *Ccl2* expression in *Rag2*^−/−^ mice in comparison with mock-treated mice. *Rag2*^−/−^ mice have smaller lymphoid organs (mature B or T lymphocytes), lack functional B and T cells, and display a stunted adaptive immune response [18]. As *Ccl2* acts to recruit immune cells to sites of inflammation after injury [25], this suggests that SARS-CoV-2 acts independently of antigen recognition produce these effects. In addition, SARS-CoV-2 (MA10) infection increased blood-brain barrier permeability, evidenced by reduced claudin 5 mRNA expression in C57BL/6, BALB/c, and *Rag2*^−/−^ mice and increased GFAP mRNA expression indicating astrogliosis, which broadly supports the hypothesis that SARS-CoV-2 induces a neuroinflammatory phenotype as found in previous studies using the hACE2 over-expressing K18 mouse model [26,27,28], rendering the brain susceptible to infection-induced dysfunction.

In our previous study, we observed that 1-year-old mice were highly susceptible to SARS-CoV-2 MA10, with high morbidity and nearly 100% mortality when infected with 10^4^ and 10^5^ PFU [16]. This might be due to SARS-CoV-2 and other emerging human coronaviruses exhibiting an age-dependent increase in disease severity [16]. Hence we aimed to determine whether increased age had any bearing on the effects on the brain of COVID infection. We observed SARS-CoV-2 (MA10) infection-induced neuroinflammation in one-year-old female BALB/c mice as shown by increased ionized calcium-binding adapter molecule 1 (Iba-1) immunoreactivity, demonstrating elevated microglial activation even at 2 days post-infection. Elevated pulmonary inflammation and tissue damage were already reported in BALB/c mice following SARS-CoV-2 (MA10) infection [16]. Further, SARS-CoV-2 (MA10) elevated perivascular accumulation of lymphocytes within the brain was consistent with an immune and inflammatory response typical of viral infection of BALB/c mice [29,30,31].

Although COVID-19-associated neurovascular manifestations are of major concern among the scientific community, only limited studies have been conducted to date in this regard. Substantial evidence of the neurological manifestation of SARS-CoV-2 infection in non-human primates has been reported recently by our group [32]. Consistent with our findings, elevated neuroinflammation, microglial activation, and perivascular cuffing were observed in the brains of aged non-human primates. In addition, Kaufer et al. demonstrated that SARS-CoV-2 infection augments microglial activation in olfactory bulbs following acute infection in Syrian golden hamsters [33]. They also found that SARS-CoV-2 infection-induced neurodegeneration 14 dpi, as demonstrated by increased hyperphosphorylated tau and alpha-synuclein in cortical neurons, underlines the long COVID-19 neurological manifestation [33].

**Figure 6:**
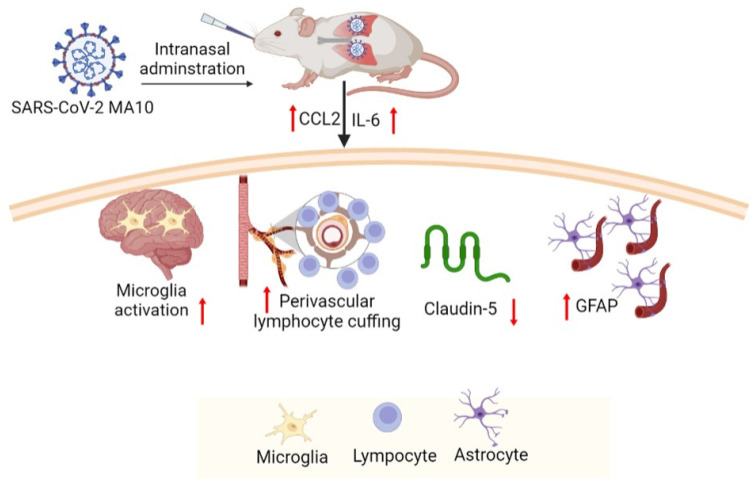
Intranasal administration of SARS-CoV-2 (MA10) upon accumulation in the mice upregulates *Ccl2* and *Il-1β*, activates microglia, induces perivascular lymphocyte cuffing, promotes astrocyte accumulation, triggers a loss of tight junction protein claudin-5. These neuroinflammatory events mediate cerebrovascular pathology in the mice infected with SARS-CoV-2 (MA10). (https://biorender.com/ accessed on 30 October 2022).

In summary, our findings demonstrate that SARS-CoV-2 (MA10) infection induces neuropathology in common laboratory mice, although it is primarily a pulmonary infection. These observations also suggest that laboratory mice infected with SARS-CoV-2 (MA10) recapitulate multiple aspects of SARS-CoV-2 mediated neuropathogenesis (Figure 6) found in humans and may serve as a suitable model to study the neurological manifestation of SARS-CoV-2 infection. Hence, the SARS-CoV-2 (MA10) infected mouse model can be used to understand neuroinflammation and associated blood-brain barrier disruption by SARS-CoV-2 infection that may have a negative impact on cognitive function. Being an initial study, our evaluations are limited to a few inflammatory mediators and histological evaluations and warrant further cross-sectional studies on pathophysiological mechanisms of SARS-CoV-2 (MA10) associated neurovascular damage. In addition, long-term experimental studies are needed to assess the consequences of SARS-CoV-2 (MA10) infection on functional neurovascular outcomes. Future studies with this model may allow for the testing of therapeutic compounds against SARS-CoV-2.

## Data Availability

Data are contained within the article.

## Supplementary Materials

The following are available online at https://www.mdpi.com/article/10.3390/v15010114/s1, Figure S1: Sub-genomic viral load in of C57BL/6, Rag^−/−^ and BALB/c mice 3 days after MA10 infection.
