# Supplementary material for: Mouse Adapted SARS-CoV-2 (MA10) Viral Infection Induces Neuroinflammation in Standard Laboratory Mice
Source: Viruses. 2022 Dec 30;15(1):114. doi: 10.3390/v15010114 (PMC9863644; doi:10.3390/v15010114)
Supplement: Supplementary file 1 [file viruses-15-00114-s001.zip › viruses-2080724-supplementary.pdf]

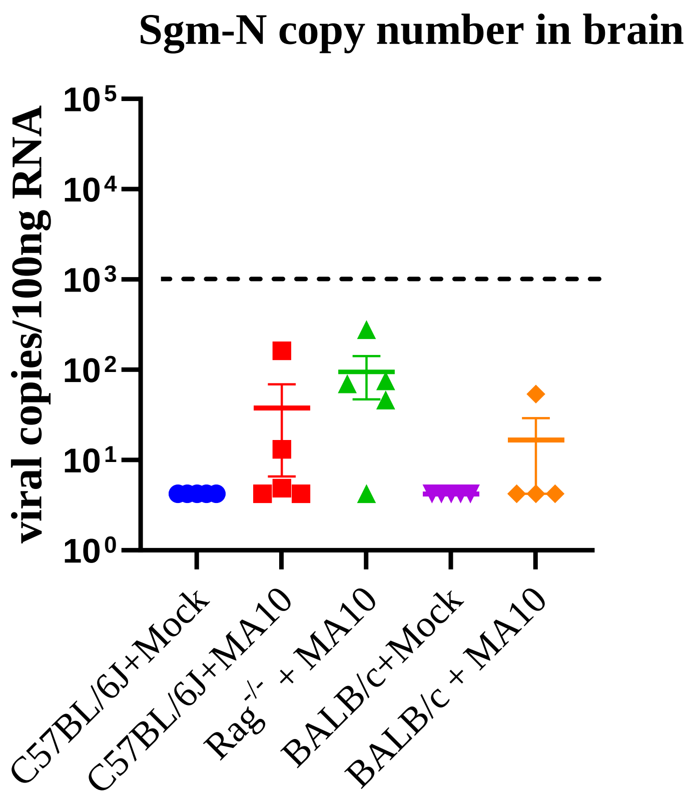

Supplementary Figure S1: Sub-genomic viral load in of C57BL/6, Rag-/- and BALB/c mice 3 days after MA10 infection. Although there is a difference in sub-genomic viral load between mock and MA10-infected animals, the difference is below the threshold detection level.
